# Unraveling mitochondrial crosstalk: a new frontier in heart failure pathogenesis

**DOI:** 10.3389/fcvm.2025.1641023

**Published:** 2025-07-15

**Authors:** Hongbo Chang, Pingge He, Weidi Liu, Hong Wu, Zhentao Wang

**Affiliations:** ^1^ Second School of Clinical Medicine, Henan University of Chinese Medicine, Zhengzhou, China; ^2^ Department of Cardiovascular Medicine, Second Affiliated Hospital of Henan University of Chinese Medicine, Zhengzhou, China

**Keywords:** mitochondria-organelle interaction, heart failure, calcium signaling, proteostasis, metabolic regulation, therapeutic target

## Abstract

Mitochondria play a central role in energy production and signal transduction in cardiomyocytes. Their dysfunction is a key contributor to the development and progression of heart failure (HF). Beyond energy metabolism, mitochondria regulate calcium homeostasis, autophagy, protein synthesis, lipid metabolism, and gene expression through close interactions with other organelles. Disruption of these interactions has been linked to HF pathophysiology.This review focuses on the dynamic communication between mitochondria and five major organelles—the endoplasmic reticulum, lysosomes, ribosomes, lipid droplets, and the nucleus. We outline how these interactions maintain cardiac homeostasis and describe how their dysfunction contributes to HF. We also highlight emerging therapeutic strategies targeting these organelle networks.

## 1 Introduction

Heart failure (HF) is one of the leading causes of hospitalization and mortality worldwide, with the prevalence continuing to rise in line with global population aging (1). Despite advances in therapeutic strategies, HF remains a significant public health issue. Imposing a substantial burden on global health and healthcare expenditures, HF urgently needs to be addressed with novel and effective treatment approaches (1).

The pathophysiological mechanisms of HF are complex and multifactorial and include metabolic dysregulation, oxidative stress, inflammatory responses, and myocardial remodeling, all of which contribute to the progressive deterioration of cardiac function (2). Mitochondria play a pivotal role in maintaining cardiomyocyte function, being responsible for generating adenosine triphosphate (ATP) through oxidative phosphorylation (OXPHOS) while regulating calcium homeostasis and redox balance. Mitochondria play a crucial role in the development and functional maturation of the embryonic heart through non-energetic metabolic processes (3). Given the heart's extremely high energy demands, mitochondrial integrity is essential for sustaining normal cardiac function, and mitochondrial dysfunction is recognized as a key pathological mechanism in the development and progression of HF. Mitochondrial dysfunction manifests as impaired oxidative metabolism, excessive production of reactive oxygen species (ROS), mitochondrial DNA damage, and activation of cell death signals (4), maladaptive changes that further accelerate the progression of HF. In addition to their autonomous functions, mitochondria establish dynamic and coordinated interactions with other organelles, enabling integrated regulation of metabolism, signaling, and cellular quality control (5–7).

Recent studies have shown that inter-organelle communication, especially the interactions between mitochondria and the endoplasmic reticulum, lysosomes, ribosomes, lipid droplets (LDs), and the nucleus, is crucial for maintaining cellular homeostasis. Disruption of these interactions is often closely associated with the onset and progression of HF. However, the spatial-temporal dynamics and specific molecular mechanisms governing these interactions under both normal and pathological conditions remain poorly understood. In this review, we aim to provide a comprehensive overview of the functional crosstalk between mitochondria and these five key organelles, focusing on their roles in maintaining cardiac homeostasis, the pathological alterations observed in HF, and potential therapeutic strategies. Furthermore, we aim to provide new insights into the mitochondria-organelle interactome and offer theoretical support for HF pathogenesis research and the development of targeted interventions by integrating the latest research advancements in this field.

## 2 Mitochondria-ER interactions: calcium signaling and energy homeostasis

### 2.1 Main forms of mitochondria-ER interactions

Mitochondria and the ER interact through a specialized physical contact site known as the mitochondria-associated ER membranes (MAMs), a structure first described in the 1950s and successfully isolated from liver tissue in the 1990s (8). MAMs are located at the interface between outer mitochondrial membranes (OMMs) and the ER, which are referred to as mitochondria-ER contact sites (MERCs). At MERCs, the physical connection between the two organelles is maintained by protein complexes, ensuring an appropriate intermembrane distance that facilitates inter-organelle communication (9). The structural dynamics of MAMs are highly adjustable and regulated by cellular metabolic and stress conditions. For example, the number of MAMs can be significantly increased by hypoxia or ER stress, and the efficiency of material exchange, such as lipid transport, is directly influenced by the width of the gap between MAMs, with tight contacts (<10 nm) being more conducive to lipid transfer (10–12). In different cell types, the proportion of the mitochondrial surface covered by MAMs typically ranges from 4% to 20% and can adapt to changes in the metabolic state. For example, in the liver after feeding, the MERC coverage can increase from 4% to 11% (13). Mass spectrometry analysis has identified approximately 1,000 proteins localized to MAMs in the brain and liver, underscoring their pivotal role in maintaining cellular homeostasis (14).

A primary role of MAMs is regulating calcium signaling to support mitochondrial energy metabolism. The IP3R–GRP75–VDAC complex facilitates the transfer of Ca^2+^ from the ER to the mitochondrial matrix via the mitochondrial calcium uniporter (MCU). IP3R1 and IP3R2 are highly enriched at MAMs and, by controlling mitochondrial calcium influx, they activate tricarboxylic acid (TCA) cycle dehydrogenases, thereby enhancing ATP synthesis (15–17).This process is further regulated by several key proteins. FUN14 domain-containing protein 1 (FUNDC1), located at MAMs, promotes calcium exchange by interacting with IP3R2 on the ER membrane, thereby maintaining MAM integrity and contributing to calcium-dependent mitochondrial bioenergetics (18–21). Sigma-1 receptor (Sig-1R), a stress-responsive chaperone, dissociates from BiP and stabilizes IP3R to enhance calcium transfer under both basal and stress conditions (22). On the ER side, sarco/endoplasmic reticulum Ca^2+^-ATPase 2a (SERCA2a) helps re-sequester calcium into the ER lumen, forming a feedback loop to maintain cytosolic and mitochondrial calcium homeostasis (23). In addition, mitochondrial calcium uptake is tightly controlled by regulatory proteins such as MICU1 and MCUb, which prevent excessive calcium influx under resting conditions. Together, these proteins ensure precise calcium signaling between the ER and mitochondria (24, 25).

MAMs are also essential metabolic hubs, coordinating lipid trafficking and synthesis. They mediate the transport of phosphatidylserine (PS) from the ER to mitochondria, where it is converted into phosphatidylethanolamine (PE), a major mitochondrial phospholipid (26, 27). MAMs also regulate cholesterol transport, cardiolipin biosynthesis, and sterol regulatory element-binding protein 1 (SREBP1)-mediated lipid synthesis, thereby contributing to mitochondrial membrane composition and function under stress conditions (28, 29).

Additionally, MAMs modulate mitochondrial dynamics to adapt to energy demands. Mitochondrial fission is initiated at MAMs via recruitment of dynamin-related protein 1 (Drp1) to mitochondrial fission 1 protein (FIS1), while fusion is regulated by mitofusin 1/2 (MFN1/2) and optic atrophy protein 1 (OPA1), which coordinate outer and inner membrane fusion, respectively (30–32). A proper balance between fission and fusion is essential for maintaining mitochondrial integrity, and its disruption is linked to mitochondrial dysfunction and cardiomyocyte damage.

Moreover, MAMs integrate endoplasmic reticulum stress (ERS) and apoptotic signaling. They are enriched with ERS sensors including BiP (binding immunoglobulin protein), IRE1α, and PERK, as well as apoptotic regulators such as Bcl-2 and Bax (32–35). Under mild stress, these pathways help restore cellular homeostasis, but prolonged activation can trigger mitochondrial Ca^2+^ overload, cytochrome c release, and apoptosis. Therefore, MAMs serve as an essential interface for the coordination of survival and death signaling in cardiomyocytes (Supplementary Figure S1).

### 2.2 Mitochondria-ER crosstalk dysregulation in HF

In heart failure (HF), the structure and function of MAMs are disrupted, leading to dysregulated calcium signaling, mitochondrial dysfunction, and cardiomyocyte injury (36). Key MAM-associated proteins show altered expression in HF. FUNDC1, which normally supports mitochondrial dynamics and calcium signaling, is often downregulated under pathological stress. Its loss disrupts interactions with IP3R2, leading to MAM dissociation, reduced mitochondrial and cytosolic Ca^2+^ levels, and impaired ATP production (18, 19). Similarly, sigma-1 receptor (Sig-1R) deficiency impairs mitochondrial calcium uptake and exacerbates cardiac remodeling (22).SERCA2a, responsible for pumping Ca^2+^ back into the ER, is frequently reduced in HF, resulting in sustained cytosolic calcium elevation and impaired ER-mitochondrial calcium cycling (23). The downregulation of OPA1, MFN1, and MFN2 further impairs mitochondrial morphology and tethering with the ER (37, 38). In parallel, deletion of Drp1 alters mitochondrial fission dynamics, leading to abnormal elongation, enhanced mitophagy, calcium overload, and ROS accumulation, which in turn trigger mPTP opening and apoptosis (39, 40). Loss of MFN2 exacerbates these effects by compromising both fusion and ER tethering (41, 42). Under conditions of prolonged ER stress, the initially protective PERK–eIF2α pathway becomes maladaptive, promoting apoptosis through CHOP activation and further exacerbated by IRE1α-mediated IP3R sensitization (43–46). Meanwhile, disruption of the balance between Bcl-2 and Bax—both enriched at MAMs—contributes to mitochondrial permeability transition and cytochrome c release, leading to cardiomyocyte death (47–50).

## 3 Mitochondria-lysosome interactions: autophagy and cardiomyocyte homeostasis

### 3.1 Main forms of mitochondria-lysosome interactions

As mitochondria and lysosomes are functionally interconnected, lysosomal dysfunction is often accompanied by mitochondrial damage. For example, defective lysosomal acidification can lead to alterations in mitochondrial dynamics and impairment of mitochondrial respiration (51, 52). Research into Pompe disease has revealed that mutations in the acid alpha-glucosidase (GAA) gene cause lysosomal glycogen accumulation, resulting in mitochondrial structural abnormalities and energy metabolism disorders, ultimately leading to hypertrophic cardiomyopathy and HF (53). This research highlights the crucial role of lysosomal integrity in maintaining mitochondrial homeostasis. Lysosome-associated proteins such as Ras-related proteins Rab5 and Rab7A, along with their respective guanine nucleotide exchange factors (GEFs), play regulatory roles in mitochondrial function (54). Conversely, the loss of mitochondrial proteins, including apoptosis-inducing factor (AIF), OPA1, PTEN-induced putative kinase 1 (PINK1), and mitochondrial transcription factor A (TFAM), compromises lysosomal activity (55).

Lysosomes primarily eliminate damaged mitochondria using two key mechanisms. One is by PINK1/Parkin-mediated mitophagy, in which damaged mitochondria are selectively recognized and degraded via autophagy receptors such as optineurin (OPTN) and nuclear dot protein 52 (NDP52) (56, 57). The other is by delivery of specific mitochondrial components by mitochondria-derived vesicles (MDVs) to lysosomes for degradation, thereby providing an alternative quality control mechanism (58).

Super-resolution electron microscopy has revealed novel forms of interaction known as mitochondria-lysosome contacts (MLCs). Approximately 15% of lysosomes form physical contact with mitochondria for durations ranging from 60 s to 13 min (59, 60). MLCs are bidirectionally regulated by components from both mitochondria and lysosomes. MLC formation is promoted by active GTP-bound Rab7, whereas the Rab7 GTPase-activating protein TBC1 domain family member 15 (TBC1D15) is recruited by FIS1 to mitochondria, facilitating Rab7 GTP hydrolysis and thereby promoting MLC dissociation (Supplementary Figure S1).

The physiological functions of MLCs include the following: (1) Regulating mitochondrial fission: MLCs mark and facilitate mitochondrial fragmentation to maintain mitochondrial dynamics and homeostasis (59). (2) Modulating lysosomal function: MLCs affect lysosomal transport and degradation capacity through Rab7 signaling (59). (3) Mediating calcium signaling: The lysosomal calcium channel transient receptor potential mucolipin 1 (TRPML1) interacts with the mitochondrial calcium channel's VDAC and mitochondrial calcium uniporter (MCU) to enhance mitochondrial oxidative phosphorylation (61).

Mitochondria-lysosome interactions form dynamic contact sites that enable the bidirectional exchange of metabolites and signaling molecules, playing a crucial role in maintaining intracellular homeostasis.

### 3.2 Mitochondria-lysosome crosstalk dysregulation in HF

Rab7 is a member of the small GTPase family that plays a crucial role in lysosome maturation, autophagosome-lysosome fusion, and endocytic pathways, including late endosome trafficking and lysosomal degradation of internalized materials. The UM-X7.1 hamster model, which simulates human dilated cardiomyopathy (DCM), experienced extensive autophagic vacuolar degeneration of cardiomyocytes along with upregulation of Rab7, ubiquitin, and cathepsin D. The disruption of the plasma membrane was compromised in these cardiomyocytes, suggesting that Rab7 may play a role in autophagy-related cell death (62). Rab7 participates in the BAG3-mediated chaperone-assisted selective autophagy (CASA), a pathway that removes damaged actin filaments and mitochondria. This process is activated by Bcl-2-associated athanogene 3 (BAG3) through stress-induced dephosphorylation, and BAG3 directly interacts with Rab7A and binds to microtubule-associated protein 1A/1B-light chain 3B (LC3B) to mediate autophagosome-lysosome fusion (63). This mechanism is particularly relevant in HF, as mutations in BAG3 lead to CASA dysregulation, resulting in protein homeostasis disruption, myocardial energy metabolism defects, and cardiomyocyte death (63).

In models of DCM and left ventricular non-compaction (LVNC) caused by gene mutations, mutations in Pleckstrin homology domain containing M2 (PLEKHM2) lead to abnormal accumulation of Rab5, Rab7, and Rab9-positive endosomes, affecting lysosomal positioning and ultimately disrupting autophagic flux (64). Rab7 dysfunction can cause the accumulation of cellular waste in cardiomyocytes, adversely affecting myocardial metabolism and contractile function and contributing to the progression of HF. Rab7 directly interacts with the mitochondrial fusion protein MFN2 to regulate the fusion of autophagosomes and lysosomes (65). Therefore, the loss of MFN2 impairs Rab7-mediated autophagic flux, leading to autophagosome accumulation, mitochondrial dysfunction, and cardiomyocyte injury (65). These findings suggest that Rab7 regulates autophagic flux and also plays a key role in maintaining mitochondrial quality control. Thus, Rab7 exerts a dual role in the pathogenesis of HF: On the one hand, it promotes lysosomal maturation and autophagic flux, helping to maintain cardiomyocyte homeostasis; on the other hand, under pathological conditions, excessive Rab7 activation may lead to autophagic cell death. The existence of this dual role suggests that modulation of Rab7 activity may represent a novel therapeutic strategy for the treatment of HF (Supplementary Table S1).

In HF, lysosomes play a crucial role in eliminating damaged mitochondria through autophagic pathways, promoting cardiac function recovery. The restoration of mitochondrial homeostasis, in turn, enhances lysosomal activity, collectively regulating the process of myocardial remodeling (66). TBC1D15 is a key regulatory factor in mitochondria-lysosome interactions. In acute myocardial infarction and ischemia-reperfusion (I/R) injury, TBC1D15 facilitates the clearance of damaged mitochondria, alleviates cardiomyocyte damage, and improves mitochondrial function (67). Moreover, TBC1D15 can directly bind to the mitochondrial fission protein Drp1, promoting the selective fission of damaged mitochondria and rendering them more susceptible to lysosomal degradation (68). This process reduces oxidative stress, inhibits cardiomyocyte apoptosis, and enhances cardiac function (68). TBC1D15-mediated mitochondria-lysosome interactions may offer therapeutic potential in cardiac protection by maintaining myocardial energy metabolism, reducing mitochondrial damage, and delaying ventricular remodeling.

In myocardial ischemia-reperfusion injury (I/R), the lysosomal Ca^2+^ channel TRPML1 becomes dysfunctional, resulting in impaired autophagic flux and hindering the effective degradation of damaged mitochondria, thereby exacerbating cardiomyocyte injury (69). Restoration of TRPML1 activity enhances lysosomal activity, promotes autophagic degradation, and facilitates mitochondrial clearance, ultimately improving cardiomyocyte survival (69). Interestingly, TRPML1 inhibition has also been shown to preserve mitochondrial function by reducing lysosomal Ca^2+^ release and decreasing oxidative stress, indicating that TRPML1 may exert bidirectional regulatory effects under different pathological conditions (70). Because patients with HF often exhibit autophagic dysfunction and mitochondrial defects, TRPML1 may play a critical role in myocardial energy metabolism, cell survival, and ventricular remodeling by modulating mitochondria-lysosome interactions.

## 4 Mitochondria-ribosome interactions: protein synthesis and myocardial injury

### 4.1 Major forms of mitochondria-ribosome interactions

Ribosomes are the exclusive sites for protein synthesis, distributed in both the cytoplasm and mitochondria. Mammalian mitochondrial ribosomes (mitoribosomes) are primarily responsible for synthesizing essential subunits required for the OXPHOS system and are therefore essential for maintaining ATP production (71). Mitochondrial proteins originate from both the nuclear genome (nDNA) and the mitochondrial genome (mtDNA). Among the proteins, mitochondrial ribosomal proteins (MRPs), which are encoded by nDNA, must be synthesized in cytoplasmic ribosomes before being imported into mitochondria to support the mitochondrial translation machinery (72, 73). MRPs are indispensable for mitochondrial function and cellular homeostasis, and their dysregulation has been closely associated with various cardiovascular diseases. For example, decreased expression of the following proteins has been linked to the development of heart disease: mitochondrial ribosomal protein S3 (MRPS3), mitochondrial ribosomal protein S22 (MRPS22), mitochondrial ribosomal protein 10 (MRP10), and mitochondrial ribosomal protein S44 (MRPS44) (74, 75). Furthermore, protein synthesis in cardiomyocytes is directly regulated by the heart-specific ribosomal gene ribosomal protein L3-like (RPL3l), and mutations in RPL3l may impair ribosomal function, ultimately leading to cardiac remodeling and myocardial injury (76, 77) (Supplementary Figure S1).

### 4.2 Mitochondria-ribosome crosstalk dysregulation in HF

Mitochondrial ribosomal protein S5 (MRPS5) is a critical component of the mitochondrial small subunit, playing a crucial role in regulating mitochondrial protein translation and maintaining myocardial homeostasis. The MRPS5 V336Y mutation leads to mitochondrial translation errors, reducing the fidelity of synthesized proteins (78). MRPS5 deficiency disrupts mitochondrial ultrastructure, impairs ATP production, and suppresses the expression of Krüppel-like factor 15 (KLF15) via the c-Myc-mediated signaling pathway, leading to metabolic dysregulation, myocardial hypertrophy, and HF (79) (Supplementary Table S1).

Ribosomal protein L3-like (RPL3l) is a muscle-specific ribosomal protein that plays a vital role in cardiac development and function. Genetic studies have shown that RPL3l variants are strongly associated with pediatric and neonatal DCM, primarily through an autosomal recessive inheritance pattern (homozygous or compound heterozygous mutations), and can lead to acute HF (80–82). Notably, RPL3l-related HF is the only known human disease linked to a tissue-specific ribosome (83). Additionally, predicted loss-of-function (pLOF) variants in RPL3l are associated with an increased risk of atrial fibrillation and cardiomyopathy, highlighting its potential role in cardiac rhythm regulation and myocardial remodeling (77).

Further research has revealed that RPL3l is regulated by myosin light chain 4 (MYL4) and succinate dehydrogenase complex flavoprotein subunit A (SDHA) and is significantly associated with immune cell infiltration, indicating that it may influence DCM progression through inflammatory mechanisms (84). RPL3l deficiency can induce compensatory upregulation of ribosomal protein L3 (RPL3), enhancing mitochondria-ribosome interactions, thereby modulating cardiac mitochondrial function and promoting ATP production (85). RPL3l also plays a crucial role in translation elongation. Its deficiency leads to increased ribosome collisions, inhibiting the synthesis of myocardial contraction-related proteins and ultimately impairing cardiac contractile function (86). These findings suggest that the pathological processes of cardiomyopathy are influenced by RPL3l through multiple mechanisms, including inflammatory regulation, mitochondria-ribosome interactions, and translational control, providing new insights into the pathogenesis of DCM and potential therapeutic strategies.

## 5 Mitochondria-LD interactions: cardiac lipid metabolism regulation

### 5.1 Major forms of mitochondria-LD interactions

Meeting the high energy demand of the heart depends on fatty acid oxidation (FAO), the primary source of ATP production. As intracellular energy storage organelles, LDs are responsible for storing and regulating neutral lipid metabolism. In recent years, the mitochondria-LD interaction has been recognized as a crucial regulator of cardiac lipid metabolism. This interaction mainly manifests in two forms: peridroplet mitochondria and LD-anchored mitochondria (87, 88). Peridroplet mitochondria are mitochondria that are somewhat separated from the LD but still maintain a close relationship, as observed in brown adipocytes, aiding in the efficient use of fatty acids (FAs). LD-anchored mitochondria are mitochondria that are highly attached to LDs and directly participate in lipid metabolism and energy supply regulation.

This interaction process is mediated by several proteins, including Plin5, mitochondria-associated GTPase 2 (MIGA2), MFN2, and tumor susceptibility gene 101 (TSG101) (89) (Supplementary Figure S1). For example, Plin5 facilitates LD expansion and FA transfer to the surface of LDs. Plin5 also localizes to mitochondria, and its overexpression enhances the association between LDs and mitochondria (90, 91). MIGA2 serves as a molecular bridge between mitochondria, the ER, and LD biogenesis (92). Interacting with Perilipin 1 (Plin1), MFN2 enhances the contact between mitochondria and LDs, promoting FA transfer and β-oxidation (93). TSG101, together with vacuolar protein sorting 13D (VPS13D), participates in the endosomal sorting complex required for transport (ESCRT) mechanism, facilitating FA transport from LDs to mitochondria and enhancing β-oxidation efficiency (94).

Additionally, AMP-activated protein kinase (AMPK), acting as an energy sensor in cells, regulates mitochondria-LD interactions under fasting conditions, promoting lipid breakdown and oxidative metabolism (95). These coordinated mechanisms ensure dynamic organelle interactions between mitochondria and LDs and adaptation to different metabolic demands.

### 5.2 Mitochondria-LD crosstalk dysregulation in HF

In HF, abnormal mitochondria-LD interactions may lead to myocardial lipotoxicity, oxidative stress imbalance, and energy metabolism disorders. Plin5 acts as a surface protein on LDs, bridging the interaction between LDs and mitochondria. Plin5 inhibits lipolysis, reducing the release of free FAs (FFAs) and preventing excessive oxidative phosphorylation of FAs in mitochondria, which otherwise contributes to ROS accumulation, thus maintaining myocardial energy homeostasis (96). Plin5 overexpression can enhance physical contact between LDs and mitochondria, reduce mitochondrial fission, and lower the rate of FA oxidation, which alleviates lipotoxic damage and decreases HF progression (97). In contrast, Plin5 deficiency accelerates lipolysis, exposing mitochondria to excessive FA load; induces oxidative stress; and promotes myocardial hypertrophy, ultimately exacerbating HF (98). Additionally, Plin5 regulates the Pirin (PIR)/nuclear factor kappa B (NF-*κ*B) axis to inhibit lipotoxicity and ferroptosis, providing further evidence of its protective role in diabetic cardiomyopathy and HF (99). Under stress conditions, PKA-mediated Plin5 phosphorylation promotes lipolysis, suggesting that Plin5 may play a bidirectional role in energy metabolism (100) (Supplementary Table S1).

However, under physiological conditions, AMPK, a central regulator of myocardial energy metabolism, activates peroxisome proliferator-activated receptor-alpha (PPAR-α) and its downstream FA oxidation-related genes, promoting FA mobilization and enhancing mitochondrial FAO capacity, thereby maintaining myocardial ATP production (101). However, during HF, reduced AMPK activity leads to decreased expression of PPAR-α and its downstream genes, limiting the heart's ability to use FA, further exacerbating energy supply deficiencies, ultimately leading to abnormal LD accumulation and lipotoxic damage, accelerating HF progression (102).

## 6 Mitochondria-nucleus interactions: metabolic and gene expression regulation

### 6.1 Major forms of mitochondria-nucleus interactions

Mitochondria-nucleus interactions play a pivotal role in regulating cellular functions, primarily through anterograde regulation and retrograde signaling, two coordinated processes that together ensure the maintenance of energy metabolism, stress responses, and mitochondrial biogenesis. Anterograde regulation refers to nuclear control over mitochondrial function and biogenesis via transcription factor activation. In response to stimuli such as cold exposure or exercise, the nucleus activates key transcription factors, including nuclear respiratory factor 1 (NRF1), nuclear factor erythroid 2-related factor 2 (NRF2), and peroxisome proliferator-activated receptors (PPARs), to regulate mitochondrial gene expression and metabolic pathways (Supplementary Figure S1). Among the actions performed by these factors, NRF1 activates mitochondrial transcription factor A (TFAM), promoting mitochondrial biogenesis (103); NRF2 regulates antioxidant defense pathways, protecting cells from oxidative stress (104); and PPARs enhance the expression of enzymes involved in mitochondrial FA oxidation, supporting ATP production and maintaining cardiac energy homeostasis (105). Additionally, the estrogen-related receptor (ERR) family (ERR-α, ERR-β, and ERR-γ) works in concert with peroxisome proliferator-activated receptor gamma coactivator 1-alpha (PGC-1α) to regulate a broad set of mitochondrial genes, enhancing mitochondrial adaptability and function (106) (Supplementary Figure S1). As a transcriptional coactivator, PGC-1α is activated by AMPK under increased energy demand (e.g., during exercise) to promote mitochondrial biogenesis and energy metabolism (107).

Retrograde signaling refers to the transmission of signals originating from mitochondria back to the nucleus, which regulates metabolic reprogramming and stress response mechanisms to prevent mitochondrial dysfunction (108). Studies have shown that mitochondria can accumulate around the nucleus to support high nuclear energy demands and accelerate retrograde signal transmission, thereby modulating mitochondrial biogenesis and autophagy (109). Under hypoxic or mitochondrial stress conditions, mitochondria cluster in perinuclear regions, enhancing ROS signaling, which affects hypoxia adaptation and cell survival (110). The translocator protein (TSPO) may act as a key scaffold protein mediating mitochondria-nuclear envelope interactions, thereby regulating cholesterol transport and nuclear transcriptional activity (111, 112).

### 6.2 Mitochondria-nucleus crosstalk dysregulation in HF

NRF1 is a key regulator of mitochondrial biogenesis, playing a crucial role in maintaining protein homeostasis and redox balance. It is particularly involved in neonatal heart regeneration and adult cardioprotection (113). Studies have shown that hypermethylation of NRF1-binding sites during HF suppresses the expression of downstream genes involved in oxidative metabolism, leading to metabolic reprogramming and acceleration of cardiac dysfunction (114). Lin28a has been found to activate the NRF1-TFAM axis, thereby improving mitochondrial function and reducing cardiomyocyte apoptosis, further underscoring the critical role of NRF1 in HF progression (115) Recent evidence also shows that NRF1 directly activates CFLAR transcription to inhibit death receptor-mediated apoptosis in cardiomyocytes under hypoxic conditions, adding to its cardioprotective repertoire (116) (Supplementary Table S1).

The Kelch-like ECH-associated protein 1 (Keap1)-Nrf2 pathway is a key regulator*y* axis in cellular defense against oxidative and electrophilic stress. Under normal conditions, Keap1 mediates the ubiquitination and degradation of Nrf2, maintaining its low basal levels (117). However, under oxidative stress or electrophilic accumulation, Keap1 undergoes conformational changes, preventing Nrf2 degradation and allowing its nuclear translocation to activate the expression of antioxidant genes (117). In both patients with HF and animal models, Nrf2 expression is generally downregulated, leading to impaired antioxidant defenses, maladaptive cardiac remodeling, and worsened cardiac function. Conversely, Nrf2 overexpression or pathway activation enhances the antioxidant capacity of cardiomyocytes, alleviates cardiac remodeling, and improves HF outcomes (118).

In HF, Nrf2 expression is regulated by multiple mechanisms, including transcriptional repression mediated by microRNAs (e.g., miR-27a, miR-28a, and miR-34a) (119, 120), direct inhibition by glycogen synthase kinase-3 (GSK-3) (118), and modulation of the Keap1-Nrf2 signaling axis by phosphoglycerate mutase family member 5 (PGAM5) (121). Additionally, cardiogenic extracellular vesicles (EVs) are enriched with Nrf2-targeting miRNAs that may act on central autonomic regulatory regions, such as the rostral ventrolateral medulla (RVLM), to enhance sympathetic nerve activity, increase cardiac workload, and further exacerbate HF progression (122).

PPARs comprise three subtypes, PPARα, PPARβ/δ, and PPARγ, which all play crucial roles in cardiac metabolic regulation. In HF, the downregulation of PPARα, which primarily regulates FAO and energy metabolism (123), impairs myocardial lipid metabolism and reduces ATP production (124). However, excessive PPARα activation may exacerbate cardiac lipotoxicity. Glycogen synthase kinase-3 alpha (GSK-3α) enhances the transcriptional activity of PPARα on lipid uptake and storage-related genes through phosphorylation at the Ser280 site, promoting myocardial lipid accumulation and diabetic cardiomyopathy (125). PPARβ/δ exerts cardioprotective effects by promoting mitochondrial biogenesis, enhancing oxidative metabolism, and upregulating antioxidant enzymes such as Cu/Zn-superoxide dismutase (Cu/Zn-SOD) and manganese superoxide dismutase (Mn-SOD), thereby mitigating oxidative stress-induced damage (126–128). PPARβ/δ activation increases mitochondrial DNA copy number, facilitates FA and glucose oxidation, and improves cardiac function under pressure overload, delaying pathological cardiac remodeling (126). In contrast, PPARβ/δ deficiency results in mitochondrial dysfunction, impaired FAO, and maladaptive cardiac metabolic remodeling, ultimately leading to myocardial hypertrophy and cardiac dysfunction (127). PPARγ primarily regulates insulin sensitivity, lipid metabolism, and inflammatory responses, playing a dual role in myocardial remodeling and HF progression (128, 167). PPARγ activation alleviates oxidative stress and myocardial fibrosis by inhibiting the NF-κB axis and transforming growth factor-beta 1/suppressor of mothers against decapentaplegic (TGF-β1/Smad) pathways, thereby improving cardiac function (129, 130). However, excessive PPARγ activation can lead to myocardial lipid accumulation, increasing cardiac workload and potentially triggering DCM (131).

ERRα and ERRγ are key regulators of cardiomyocyte maturation, acting as transcriptional activators of metabolic and structural genes in the adult heart (106). Cardiac-specific overexpression of ERRγ induces cardiomyocyte hypertrophy, increased cell death, and fibrosis, ultimately leading to HF (132). In patients with chronic congestive HF, ischemic HF, and idiopathic end-stage HF, ERRα and its target genes are significantly downregulated.

A master regulator of mitochondrial biogenesis, PGC-1α is activated through phosphorylation by AMPK and deacetylation by sirtuin 1 (SIRT1), These modifications cooperatively enhance PGC-1α activity, thereby enhancing mitochondrial gene expression mediated by NRF1, NRF2, and ERRα. This PGC-1α–mediated pathway improves cardiac energy metabolism and reduces ROS levels, exerting cardioprotective effects (133). Gene therapy with adeno-associated virus (AAV)-mediated anti-miR-199a upregulates the PGC-1α/ERRα axis, restores mitochondrial function, and alleviates cardiac hypertrophy (134). Additionally, overexpression of C1q/tumor necrosis factor-related protein 5 (CTRP5) activates the AMPKα2 signaling pathway, leading to increased PGC-1α expression, reduced ischemia-reperfusion injury, and improved infarct-induced HF outcomes (135).

## 7 Therapeutic strategies targeting organelle crosstalk in HF

### 7.1 Targeting mitochondria-ER crosstalk for therapeutic intervention

Intervention strategies targeting MAMs dysfunction have emerged as a crucial research direction in HF treatment. For example, the Danqi Pill has been shown to regulate the coordination between unc-51-like autophagy activating kinase 1 (ULK1) and PGAM5, thereby enhancing FUNDC1-mediated mitophagy, potentially influencing mitochondria-ER interactions and preserving myocardial energy metabolism (136). Moxibustion therapy upregulates OPA1 expression while reducing DRP1 and FIS1 levels, thereby inhibiting excessive autophagy and suppressing doxorubicin (DOX)-induced FUNDC1 signaling, ultimately mitigating myocardial injury (137). Treatment with the antioxidant α-lipoic acid (α-LA) alleviates pressure overload-induced ventricular remodeling via a FUNDC1-dependent mechanism (138).

Selective serotonin reuptake inhibitors (SSRIs), such as fluvoxamine, exert cardioprotective effects by activating the Sig-1R and its downstream Akt-endothelial nitric oxide synthase (eNOS) signaling pathway, improving cardiac dysfunction induced by transverse aortic constriction and pressure overload (139). Mitochondrial division inhibitor ameliorates HF by preventing excessive mitochondrial fission and mitophagy (140). Fenofibrate protects the myocardium from hypertension-induced remodeling by maintaining the balance of MFN2, DRP1, and Parkin, thereby stabilizing mitochondria-ER contact sites (141).

Moreover, resveratrol has been shown to activate the SIRT1/MFN2 axis, improving DOX-induced mitochondrial dysfunction, reducing cardiomyocyte apoptosis, and stabilizing mitochondria-ER interactions, alleviating mitochondrial stress and energy metabolism disorders (142). Ferulic acid, astragaloside, and tyrosol mitigate cardiomyocyte apoptosis by inhibiting excessive activation of the PERK/eIF2α/activating transcription factor 4 (ATF4)/CHOP pathway, alleviating ERS-induced cardiac damage (143). Additionally, left ventricular assist device (LVAD) therapy reverses the imbalance of the Bcl-2/Bax ratio in patients with HF, supporting the pathological role of MAMs in HF progression (144) (Supplementary Table S1).

### 7.2 Therapeutic implications of mitochondria-lysosome crosstalk in autophagic regulation

Research has demonstrated that administration of factors targeting Rab7 regulation can ameliorate the pathological progression of HF. Administration of granulocyte colony-stimulating factor (G-CSF) can significantly reduce Rab7-associated autophagic activity, decreasing cardiomyocyte death and improving both cardiac function and survival rates (62). Administration of resveratrol exerts cardioprotective effects by activating the SIRT1/forkhead box O1 (FOXO1)/Rab7 axis, promoting autophagic flux, alleviating oxidative stress-induced damage, and improving cardiac function in diabetic cardiomyopathy models (145). Administration of ferulic acid provides significant cardioprotective effects in hypoxia/reoxygenation (H/R) injury models by inhibiting PTEN-induced kinase 1 (PINK1)/Parkin-dependent mitophagy, thereby reducing mitochondrial-lysosomal interactions, mitigating H/R-induced cardiomyocyte apoptosis, and preserving mitochondrial function (146) (Supplementary Table S1).

### 7.3 Restoring mitochondria-ribosome coordination to enhance cardiac protein homeostasis

Targeting MRPs and cardiac-specific ribosomal factors, such as RPL3l, may offer novel therapeutic strategies for HF. For example, administration of exogenous Klf15 has been shown to partially reverse MRPS5 deficiency-induced cardiac dysfunction, suggesting that modulation of the MRPS5-Klf15 axis may help correct myocardial metabolic abnormalities and delay HF progression (79). MYL4 and SDHA have been identified as upstream regulators of RPL3l, highlighting the potential of targeting the MYL4-SDHA-RPL3l axis as a new strategy for mitigating cardiac remodeling and treating HF (84) (Supplementary Table S1).

### 7.4 Modulating mitochondria-LD interactions to restore cardiac lipid metabolism

Therapeutic strategies targeting mitochondrial-LD interactions, with a focus on optimizing LD metabolism, reducing lipotoxicity, and mitigating oxidative stress, have emerged as novel approaches for HF intervention. Acetylcholine (ACh) enhances mitochondrial-LD interactions by upregulating Plin5, thereby promoting LD lipolysis and reducing cardiomyocyte apoptosis (147). Metformin, resveratrol, and exercise regulate Plin5 expression, optimize FA metabolism, decrease cardiac LD accumulation, and improve energy homeostasis (148). In addition, a randomized controlled trial in nondiabetic HFrEF patients demonstrated that metformin significantly increased total antioxidant capacity and attenuated left ventricular remodeling, supporting its potential cardioprotective role beyond glycemic control (149). Maintaining an optimal LD reservoir is a crucial strategy for preventing oxidative stress, as excessive lipolysis can trigger ROS release and exacerbate myocardial injury. N-acetylcysteine (NAC), a precursor of glutathione, reduces oxidative stress and improves cardiac function (96). Moreover, cardiac contractility modulation, a non-pharmacological intervention, can enhance myocardial energy metabolism via the AMPK-PPAR-α axis, reducing abnormal LD and glycogen accumulation while increasing ATP production. This effect is likely associated with AMPK-mediated promotion of mitochondrial-LD contacts and enhancement of FA oxidation capacity (102) (Supplementary Table S1).

### 7.5 Targeting mitochondria-nucleus communication for metabolic and gene regulation

Various pharmacological agents can ameliorate HF progression by modulating NRF1 and related pathways. For example, the Danqi Pill enhances glucose metabolism and mitochondrial function via the HIF-1α/PGC-1α pathway, improving myocardial energy supply and HF outcomes (150). Perindopril and carvedilol activate the PGC-1α/NRF1/TFAM axis, promoting mitochondrial biogenesis, enhancing antioxidant capacity, and increasing ATP production to improve cardiac function (151, 152).

Recent studies have extended these findings to HFpEF, a major HF subtype with distinct metabolic dysregulation. In mouse models, berberine improved cardiac function by restoring mitochondrial homeostasis and reducing apoptosis through AMPK/PGC-1α signaling, accompanied by NRF1 and TFAM upregulation (153). Similarly, hydrogen sulfide (H₂S) alleviated diastolic dysfunction by activating the PGC-1α/NRF1/TFAM axis and correcting mitochondrial abnormalities, whereas genetic CSE deletion aggravated these defects but could be rescued by NaHS or the PGC-1α activator ZLN005 (154).

Targeting the Keap1-Nrf2 pathway has also emerged as a crucial therapeutic strategy for HF. Tanshinone IIA sulfonate (TIIA) interacts hydrophobically with Keap1, facilitating its dissociation and degradation, thereby upregulating Nrf2 transcription and reducing H₂O₂-induced cardiomyocyte apoptosis (155), a meta-analysis of 14 RCTs confirmed its clinical efficacy as an adjunctive HF therapy (156). Puerarin activates the Nrf2/ROS pathway, downregulates Keap1 expression, and promotes Nrf2 nuclear translocation, ultimately attenuating myocardial fibrosis (157). Additionally, the Yiqi Huoxue Recipe improves mitochondrial membrane potential and reduces cardiomyocyte apoptosis through the Keap1/Nrf2/HIF-1α axis, mitigating myocardial injury in HF models (158).

As a precursor of flavin adenine dinucleotide (FAD), riboflavin activates the SCAD-DJ-1-Keap1-Nrf2 pathway, reduces oxidative stress, and enhances cardiac function (159). LingGui-Zhu-Gan Decoction (LGZGD) has been shown to attenuate oxidative damage and cardiomyocyte apoptosis via the Nrf2/Keap1/HO-1 signaling pathway (160). PPARα agonists, such as fenofibrate, have been shown in the ACCORD Lipid trial to reduce heart failure hospitalization or cardiovascular death in patients with type 2 diabetes (HR = 0.82, *P* = 0.048). A Korean nationwide cohort study further confirmed a lower risk of HF hospitalization with fenofibrate use (HR = 0.907) (161, 162). Statins such as atorvastatin exert cardioprotective effects by inhibiting the advanced glycation end-products-receptor for advanced glycation end-products-extracellular signal-regulated kinase 1/2 signaling pathway through PPARγ, thereby reducing myocardial fibrosis (130).

A multicenter, randomized, double-blind clinical trial demonstrated that Qili Qiangxin Capsules significantly reduced NT-proBNP levels, improved cardiac function, and slowed adverse cardiac remodeling in patients with chronic HF (163). Laboratory studies further revealed that Qili Qiangxin Capsules upregulate PPARγ, alleviating post-myocardial infarction cardiac remodeling, preserving cardiac function, and reducing apoptosis and fibrosis (164). Xin-shu-bao tablets also exert cardioprotective effects in HFrEF by regulating PPARγ/MFGE8-mediated lipid metabolism and attenuating ventricular remodeling (165).Together, these findings underscore the potential of multi-target traditional therapies to modulate mitochondrial–nuclear crosstalk and lipid homeostasis in subtype-specific HF management (Supplementary Table S1).

## 8 Concluding remarks

Current research has revealed the key role of organelle interactions, particularly involving mitochondria, in maintaining cellular homeostasis and contributing to the pathogenesis of heart failure (HF). However, several important questions remain unresolved. One major gap is the lack of systematic understanding of how the spatiotemporal characteristics of mitochondrial-organelle crosstalk evolve across different HF subtypes and disease stages. Notably, emerging clinical and experimental evidence suggests that mitochondrial dynamics, calcium signaling, and metabolic coupling via MAMs or other contact sites are not uniformly altered in HF but differ significantly between HF with preserved ejection fraction (HFpEF) and reduced ejection fraction (HFrEF). For example, HFpEF is often associated with enhanced MAMs formation and increased mitochondrial calcium influx, while HFrEF typically exhibits MAMs disruption and mitochondrial calcium depletion—highlighting the need for subtype-specific mechanistic investigation (166).

Moreover, conflicting findings persist regarding the role of specific regulators such as MFN2 or FUNDC1 in different experimental models, reflecting variability in disease modeling strategies and technical approaches. To address these inconsistencies, future studies should prioritize the use of clinically relevant models and human myocardial samples to dynamically profile mitochondrial-organelle interactions throughout disease progression and therapeutic intervention. This will help clarify whether these interactions are causative, compensatory, or maladaptive at different HF stages.

Furthermore, research should aim to develop precision strategies targeting organelle interactions—for instance, enhancing specific tethering proteins or modulating inter-organelle calcium exchange—to restore myocardial metabolic homeostasis and prevent maladaptive remodeling. With the advancement of tools such as single-cell multi-omics, high-content imaging, and mitochondrial proximity labeling, it will become increasingly feasible to decode the organelle interactome with high spatiotemporal resolution.

In conclusion, achieving a deeper and more differentiated understanding of mitochondrial-organelle interactions across HF phenotypes will provide a mechanistic foundation for the development of targeted, personalized therapeutic approaches—offering new hope for improving prognosis and treatment response in this heterogeneous disease.
