# Supplementary material for: Unraveling mitochondrial crosstalk: a new frontier in heart failure pathogenesis
Source: Front Cardiovasc Med. 2025 Jul 15;12:1641023. doi: 10.3389/fcvm.2025.1641023 (PMC12303913; doi:10.3389/fcvm.2025.1641023)
Supplement: Supplementary file 1 [file Datasheet1.docx]

Supplementary Material

#
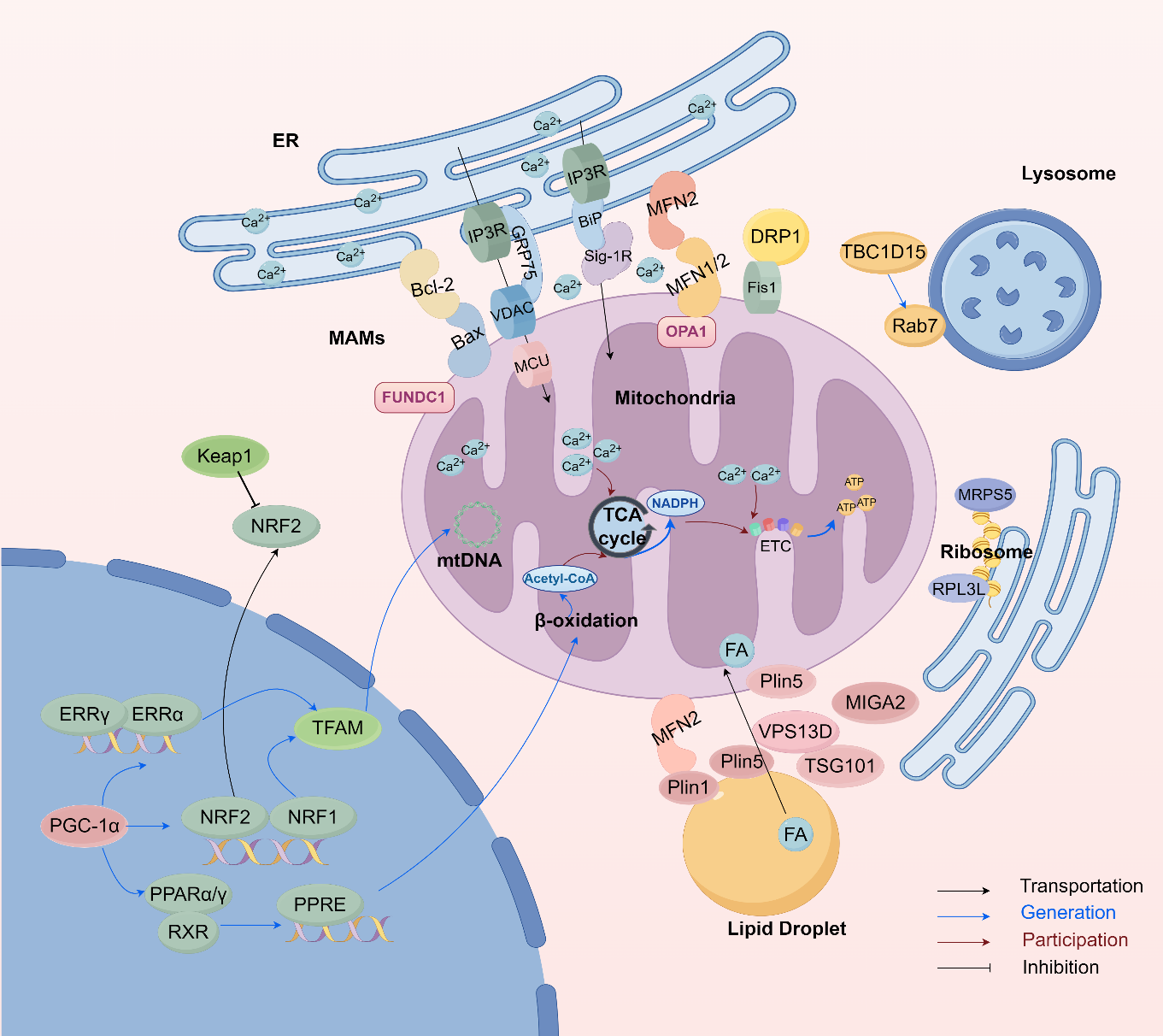
Supplementary Figures

**Supplementary Figure 1.** **Mitochondrial interactions with intracellular organelles and regulatory pathways involved in heart failure.** This diagram illustrates the structural and functional interactions between mitochondria and major intracellular components, including the endoplasmic reticulum (ER), lysosome, ribosome, lipid droplets, and nucleus, highlighting key molecular players and signaling pathways involved in mitochondrial homeostasis and energy metabolism in cardiomyocytes. **Mitochondria-associated ER membranes (MAMs):** IP3R-GRP75-VDAC complex mediates Ca²⁺ transfer from the ER to mitochondria. Sig-1R, Bcl-2, Bax, and PERK regulate MAM stability, calcium homeostasis, and stress responses. FUNDC1 and OPA1/MFN1/2 modulate mitochondrial dynamics and mitophagy. **Lysosome interaction:** Rab7 and TBC1D15 coordinate lysosomal degradation of damaged mitochondria via mitophagy. **Ribosome interaction:** MRPS5 (mitochondrial ribosome) and RPL3L (cytosolic ribosome) mediate protein synthesis essential for mitochondrial function. **Lipid droplet interaction:** Plin5, MFN2, MIGA2, VPS13D, and TSG101 regulate lipid transfer and β-oxidation, contributing to fatty acid metabolism and energy production. **Mitochondria-nucleus signaling:** Transcriptional coactivator PGC-1α, along with NRF1, NRF2, TFAM, ERRα/γ, and PPARs, promotes mitochondrial biogenesis and oxidative metabolism. PPARα/γ-RXR complex: A nuclear transcription factor complex that binds to peroxisome proliferator response elements to regulate genes involved in lipid metabolism and energy homeostasis. PPRE/DNA: Peroxisome proliferator response element, a specific DNA sequence located in the promoter region of target genes that is recognized and bound by PPAR-RXR complexes to initiate transcription. ERRα/γ, NRF1: Nuclear transcription factors that regulate mitochondrial biogenesis by activating TFAM. Keap1-NRF2 axis controls antioxidant gene expression in response to oxidative stress. **Arrows:** Black arrows indicate molecular transport or translocation. Blue arrows indicate generation or synthesis (e.g., ATP and NADPH). Brown arrows indicate pathway participation or functional involvement. Black T-shaped arrows indicate inhibition or negative regulation.

# Supplementary Table

**Table 1. Key proteins involved in organelle interactions and their roles in heart failure**

| **Interaction type** | **Key proteins** | **Function** | **Role in heart failure** | **Potential therapeutic strategy** |
| --- | --- | --- | --- | --- |
| **Mitochondria-ER** | IP3R-GRP75-VDAC | Mediates calcium transfer from ER to mitochondria; maintains contact site integrity | Disrupts calcium balance, impairs cardiac function | Targeting this complex can restore calcium homeostasis |
|  | FUNDC1 | Regulates mitophagy and mitochondria–ER contacts | Dysfunction leads to calcium imbalance and energy defects | Danqi Pills and moxibustion therapy improve FUNDC1-mediated mitophagy and protect cardiac function |
|  | Sigma-1R | Stabilizes ER–mitochondria contact and reduces ER stress | Loss increases oxidative stress and worsens HF | Fluvoxamine (selective serotonin reuptake inhibitor, SSRI) activates Sig-1R |
|  | OPA1 | Supports mitochondrial fusion and membrane structure | Loss impairs metabolism and increases injury | Boosting OPA1 may protect mitochondria |
|  | MFN1/2 | Promotes ER–mitochondria tethering and fusion | Downregulation damages mitochondrial dynamics | Targeting MFN1/2 may restore mitochondrial function and alleviate myocardial injury |
|  | PERK | Initiates ER stress response and limits protein overload | PERK activation inhibits protein synthesis and regulates calcium signaling; excessive activation increases cardiomyocyte apoptosis | Ferulic acid, *Astragalus* polysaccharides, and tyrosol modulate PERK signaling to mitigate heart failure progression |
|  | Bcl-2/Bax | Balances cell survival and apoptosis | Bcl-2/Bax imbalance leads to mitochondrial Ca²⁺ overload and mPTP opening, inducing cardiomyocyte apoptosis | Left ventricular assist device therapy can restore Bcl-2/Bax ratio and improve cardiac function |
| **Mitochondria-lysosome** | Rab7 | Drives autophagosome–lysosome fusion | Rab7 dysfunction impairs autophagy, reduces mitochondrial quality control, and exacerbates myocardial damage | G-CSF inhibits Rab7-related autophagy, reducing cell death; resveratrol activates the SIRT1/FOXO1/Rab7 axis, alleviating oxidative stress |
|  | TBC1D15 | Regulates Rab7 activity and mitochondrial clearance | TBC1D15 dysfunction may dysregulate Rab7, affecting mitochondrial degradation and promoting myocardial damage | Targeting TBC1D15 may restore Rab7 function and improve mitochondrial quality control |
|  | TRPML1 | Controls lysosomal calcium signaling | TRPML1 dysfunction impairs lysosomal function, affecting mitochondrial quality control and autophagy, increasing oxidative stress | TRPML1 agonists (such as those alleviating oxidative stress) can restore lysosomal function and reduce myocardial damage |
| **Mitochondria-ribosome** | MRPS5 | Supports mitochondrial protein synthesis | MRPS5 deficiency leads to insufficient energy supply in cardiomyocytes, triggering heart failure | Exogenous Klf15 can partially reverse MRPS5 deficiency and improve mitochondrial function |
|  | RPL3L | Controls muscle-specific protein translation | RPL3L dysfunction may impair myocardial adaptation and cause pathological cardiac remodeling | Targeting the MYL4–SDHA–RPL3L axis to mitigate cardiac remodeling in HF |
| **Mitochondria-lipid Droplet** | Plin5 | Bridges lipid droplets and mitochondria for fatty acid use | Plin5 downregulation leads to lipid accumulation, lipotoxicity, and increased oxidative stress | Acetylcholine, metformin, and resveratrol upregulate Plin5, optimizing fatty acid metabolism |
|  | AMPK | Enhances energy metabolism via lipid oxidation | AMPK inactivation leads to lipid accumulation and myocardial energy imbalance | Exercise and CCM therapy activate the AMPK-PPAR-α axis to enhance fatty acid oxidation and ATP production |
| **Mitochondria-nucleus** | NRF1 | Activates mitochondrial gene expression | NRF1 downregulation reduces antioxidant capacity, impairs ATP production, and promotes heart failure | Danqi Pills, perindopril, and carvedilol activate the PGC-1α/NRF1/TFAM axis to enhance mitochondrial function |
|  | Keap1-Nrf2 | Protects cardiomyocytes via antioxidant stress responses | Reduced Nrf2 activity leads to oxidative stress accumulation and exacerbates myocardial damage | Yiqi Huoxue Recipe, riboflavin, and Ling-Gui-Zhu-Gan Decoction activate the Nrf2 pathway |
|  | PGC-1α | Promotes mitochondrial biogenesis and energy metabolism | PGC-1α downregulation impairs mitochondrial function and increases oxidative stress, promoting heart failure | Perindopril and carvedilol activate the PGC-1α/NRF1/TFAM axis; Danqi Pills enhance mitochondrial function via the HIF-1α/PGC-1α pathway |
|  | PPARα/β/δ/γ | Regulates metabolism and cardiac energy supply | PPAR dysfunction causes metabolic disorders | PPARα agonists (e.g., fenofibrate) improve lipid metabolism and reduce triglyceride levels, decreasing cardiovascular events; atorvastatin inhibits AGEs-RAGE-ERK1/2 signaling via the PPARγ pathway to reduce myocardial fibrosis; and Qili Qiangxin Capsules upregulate PPARγ, improving cardiac function and attenuating adverse remodeling |
|  | ERRα/γ | Regulates mitochondrial metabolic genes and maintains cardiac energy balance | ERRα/γ downregulation weakens ATP production | Targeting ERRα/γ improves mitochondrial metabolism and energy homeostasis in HF |
